# Impact of antiretroviral therapy on fertility desires among HIV-infected persons in rural Uganda

**DOI:** 10.1186/1742-4755-8-27

**Published:** 2011-10-06

**Authors:** Walter Kipp, Jennifer Heys, Gian S Jhangri, Arif Alibhai, Tom Rubaale

**Affiliations:** 1 Department of Public Health Sciences, School of Public Health, University of Alberta, Edmonton, Alberta, Canada; 2 Kabarole District Health Department, P.O. Box 27, Fort Portal, Uganda

**Keywords:** highly active antiretroviral therapy, fertility desires, family planning, HIV/AIDS, knowledge, mother-to-child-transmission, peri-natal transmission, resource-limited setting, Uganda

## Abstract

**Background:**

Little is known about the fertility desires of HIV infected individuals on highly active antiretroviral therapy (HAART). In order to contribute more knowledge to this topic we conducted a study to determine if HIV-infected persons on HAART have different fertility desires compared to persons not on HAART, and if the knowledge about HIV transmission from mother-to-child is different in the two groups.

**Methods:**

The study was a cross-sectional survey comparing two groups of HIV-positive participants: those who were on HAART and those who were not. Semi-structured interviews were conducted with 199 HIV patients living in a rural area of western Uganda. The desire for future children was measured by the question in the questionnaire "Do you want more children in future." The respondents' HAART status was derived from the interviews and verified using health records. Descriptive, bivariate and multivariate methods were used to analyze the relationship between HAART treatment status and the desire for future children.

**Results:**

Results from the multivariate logistic regression model indicated an adjusted odds ratio (OR) of 1.08 (95% CI 0.40-2.90) for those on HAART wanting more children (crude OR 1.86, 95% CI 0.82-4.21). Statistically significant predictors for desiring more children were younger age, having a higher number of living children and male sex. Knowledge of the risks for mother-to-child-transmission of HIV was similar in both groups.

**Conclusions:**

The conclusions from this study are that the HAART treatment status of HIV patients did not influence the desire for children. The non-significant association between the desire for more children and the HAART treatment status could be caused by a lack of knowledge in HIV-infected persons/couples about the positive impact of HAART in reducing HIV transmission from mother-to-child. We recommend that the health care system ensures proper training of staff and appropriate communication to those living with HIV as well as to the general community.

## Background

The decision whether or not to have children is often complex and influenced by many factors. HIV-positive individuals in Africa have additional considerations to take into account when deciding whether or not to have children. These include the possibility of passing HIV from mother-to-child and the likelihood that one or both parents could die prior to the child reaching adulthood [1]. Mother-to-child transmission (MTCT) of HIV happens in either of two ways: through perinatal infection and through breastfeeding. Regardless of these concerns, many Africans after receiving a positive HIV diagnosis still elect to have children for various personal, cultural and economic reasons [2]. In most African societies a common expectation of marriage is that the couple will have children [3]. This is an especially important expectation in Uganda as children become members of the paternal clan [4]. In African societies women are often valued by their ability to bear children and a very high social good is placed on fertility; therefore, the pressure on women to have children is very high [5].

The birth of an HIV-positive child to a HIV-positive parent or couple is a situation faced by many families in sub-Saharan African countries. However, the advent of available highly active antiretroviral therapy (HAART) in many sub-Saharan African countries has changed the situation for HIV-positive persons. HAART has been shown to drastically reduce the risk of MTCT of HIV: The risk of MTCT in Denmark was less than 1% in all HIV-positive women (all on HAART) who gave birth between 2000-2005, and 1.7% in a large reference center for pregnant HIV-positive women in Belgium [6,7]. Similarly, HAART has been found to reduce the risk of vertical transmission of HIV in sub-Saharan African countries. For example, the risk of MTCT of HIV in women from a general sample from several sub-Saharan African countries decreased from 30.9% in untreated women to 4.9% in women on HAART [8]. Similarly, in Botswana, the number of new pediatric HIV infections has dropped from 4,600 in 1999 to 890 in 2007 due to nearly complete coverage of an effective antiretroviral treatment program [9]. In Cote d'Ivoire, the MTCT rate of HIV was 5.6% in formula-fed infants and 6.8% in infants with short-term breastfeeding reported by mothers on HAART [10]. These figures show the vast benefits of HAART in reducing the risk of vertical transmission of HIV. Some studies from sub-Saharan Africa have shown that an HIV diagnosis causes people generally to choose to have fewer children [3,11,12]. Other research has shown that HIV infection does not have a marked impact on fertility decisions, particularly for those who do not show signs or symptoms of disease [2,13,14].

Based on many reports indicating that HAART provision significantly decreases the risk of vertical transmission of HIV, one could expect that HIV-positive mothers/couples on HAART in sub-Saharan African countries would by now be more likely to opt to have children. It is therefore important to investigate if voluntary testing and counseling (VCT) for HIV infection and programs to prevent mother-to-child transmission (PMTCT) of HIV services are effective in counseling their clients on these recent findings and benefits of HAART. One study from South Africa stated that HAART increased the fertility desire in couples over time [15]. Similarly, a pan African study from seven countries showed that the pregnancy rate of HIV-positive women on HAART was significantly higher compared to those not on HAART [16]. A Zimbabwean study found that women on HAART were more likely to plan for a future child, but some women still had doubts about the effectiveness of HAART to prevent mother-to-child transmission of HIV [17].

Ugandan studies on the association between HAART and fertility revealed mixed results. One study from rural south-western Uganda showed that HIV-positive women on HAART had an increased desire for children but did not experience an increase in actual fertility [18]. Similarly, another study from rural eastern Uganda showed that HAART was not associated with pregnancy [19]. A survey of women attending Mbarara hospital in south-western Uganda revealed that HIV-positive women had a lower desire for future children irrespective of their HAART treatment status than HIV-negative women [20]. Another study from Mbarara district in south-western Uganda, found that HIV-positive women on HAART were more than three times likely to use contraception compared to those not on HAART [21]. In contrast to these findings, one study from Rakai district in central Uganda described a significant positive association between HAART treatment and becoming pregnant in 712 women attending a hospital [22].

The participants in the above mentioned studies included mostly only HIV-positive women, that is, HIV-positive men were not interviewed. Male involvement in reproductive health and fertility has long been recognized as important, but has not been achieved in a tangible way in many sub-Saharan African countries [23-25]. In order to have a balanced view on whether being on HAART changes fertility desires of individuals and couples, it is crucial to include both sexes. We interviewed male and female HIV-positive participants on this issue. The objectives of the study were:

1. To investigate whether HIV-positive persons on HAART had different fertility desires compared to HIV-positive persons not on HAART and whether these were different between men and women;

2. To assess the knowledge of mother-to-child transmission of HIV in participants on HAART and not on HAART.

The study took place from September to December 2006 in two districts, Kabarole and Kamwenge, in western Uganda. Other results from the main quantitative component of this study on fertility and HIV status are published elsewhere [26].

## Methods

### Study setting

Participants were recruited through correct health centres located in Rwimi and Kibiito sub-counties in Kabarole District, and the Bigodi sub-county in Kamwenge District. The government-run health centers were located along two major roads and offered clinical and public health services, as well as VCT programs to prevent mother-to-child transmission of HIV. The Rwimi and Kibiito Health Centres also offered free HAART for eligible HIV patients (CD4 cell count < 200 cells/ml and/or WHO stage 3 and higher). The area surrounding these health centres has a predominantly agricultural-based economy and subsistence farming is the main occupation. This area is very fertile, allowing for high yields of basic food crops such as maize, cassava, cooking banana and beans.

### Study design

The study applied a cross-sectional, quantitative design. We report the findings based on a survey which used a structured questionnaire administered through interviews to gather data from participants.

### Recruitment of participants

The study inclusion criteria were: age 18-44 years, married or cohabitating with a partner and having a positive HIV test result. Persons who were bedridden were excluded from the study. All HIV-positive persons registered at two health centers (Rwimi and Kibiito) were invited to participate in the study. In order to increase the sample size, all HIV-positive individuals of an HIV patient support group in Bigodi sub-county were also included in the study. Individuals identified were contacted by health care workers and asked to participate in the study. Ninety-two percent of eligible patients agreed to be interviewed. Participants had the option of being interviewed either in their home or at their local health centre. Of the 199 persons who participated in the study, 122 individuals were on HAART and 77 were not on HAART. The HAART status was derived from interviews and verified using health records.

### Data collection and analysis

A questionnaire was developed in consultation with local experts to assess the socio-demographic characteristics of the study participants. Most questions used were derived from published sources and were already tested for its reliability and validity, e.g. questions derived from the Ugandan Demographic and Health Survey [27]. The questionnaire included questions related to HIV testing, HIV status and fertility desires, treatment status (HAART or no HAART), attitudes towards childbearing of HIV-infected women/couples and risk perceptions of MTCT of HIV. The questionnaire was translated into the local language, Rutooro, and back translated into English for linguistic reliability. It was pre-tested in the study area by 15 persons not part of the study. The instruments' reliability was assessed through a test-retest exercise of 26 randomly selected participants seven days after the questionnaire was first administered. The overall percent agreement obtained in the re-test was 92.4%. For the most important question referring to the main study outcome variable "Do you want more children in future" agreement was 96.2%. (For those participants who reported to be pregnant at the time of the interview this question was phrased as "Do you want more children in future in addition to the current pregnancy"). The final questionnaire was administered by trained interviewers in the local language. The interviews lasted around 40 minutes.

All of the interview data was entered into Microsoft Access and then transferred into STATA for statistical analysis [28]. A p value of < 0.05 was considered statistically significant (all statistical tests were two sided). Data from open-ended survey questions was coded and analyzed using descriptive statistics. The chi-square test and t-test were used for bivariate data analysis. Bivariate and multivariate logistic regression was used to examine and model the variable "Desire to have more children in future" with a binary outcome (yes/no) and HAART status as the main covariate of interest. Independent variables included demographic and socio-economic characteristics as well as various HIV-related factors such as the HIV-serostatus of the respondent's partner, experience of any AIDS-related symptoms or illness, and experience of an AIDS-related death (either within their family or of a child in their community). All the independent variables that were significant at p < 0.2 in bivariate analyses or important confounding variables (e.g. sex) were selected and fit into a multivariate model. Variables found to be statistically significant in the multivariate model (p < 0.05) were kept in the final model.

### Study approval and ethical considerations

Ethics approval was provided by the University of Alberta's Health Research Ethics Board Panel B. In Uganda, approval for the study was obtained from the Uganda National Council of Science and Technology, Kampala. The study was also approved by the District Health Officers of Kabarole and Kamwenge districts, and the political representatives of the study areas. Each participant was informed about the study through an information letter that was read to each participant. All participants signed a consent form.

## Results

### Demographic and socio-economic characteristics of study participants

One hundred twenty two (61.3%) of the study participants were female which is representative of this clinic population. The mean age of males was 35.7 years (SD 5.7 years, range 20-44 years) while the mean age of females was 33.3 years (SD 6.0 years, range 18-44 years). This age difference was statistically signficant (p = 0.006). One-hundred and twenty two (61.3%) participants were currently on HAART while 77 (38.7%) were not. More female participants were on HAART compared to males (41.8% vs. 33.8%, p = 0.257). Both female and male participants on HAART were younger compared to those not on HAART (female: 31.1 vs. 34.8 years, p < 0.001; male: 33.1 vs. 37.0 years, p = 0.004). Five participants (6.5%) in the HAART group reported to be pregnant during the survey, compared to three (2.5%) in the non-HAART group. The majority of the study respondents were subsistence farmers with a primary education, living in metal-roofed mud huts. The participants had a wide range of tribal and religious affiliations. For more details see Table 1:

**Table 1:** Demographic characteristics and antiretroviral treatment status of survey respondents (n = 199)

Variable	N	% Want More Children	**p-value**^a^
Sex			0.131
Female	122	10.7	
Male	77	18.2	
Marital status			0.870
Married	109	12.8	
Cohabiting	76	13.6	
Occupation			0.430
Farmer/peasant	132	11.4	
Businessperson	26	19.2	
Other	41	17.1	
Education			0.583
None	36	16.7	
Lower Primary	61	11.5	
Upper Primary	74	16.2	
Secondary and above	28	7.1	
Dwelling quality^b^			0.366
Low	17	23.5	
Medium	158	13.3	
High	24	8.3	
Ownership of Radio			0.918
No	50	14.0	
Yes	149	13.4	
Ownership of Bicycle			0.718
No	134	14.2	
Yes	65	12.3	
Ownership of Animals			0.182
No	87	17.2	
Yes	112	10.7	
Religious affiliation			0.911
Catholic	92	12.0	
Protestant	76	15.6	
Muslim	8	12.5	
Other	23	13.0	
Ethnic group			0.043
Mutooro	112	8.9	
Mukiga	56	16.1	
Other	31	25.8	
ARV treatment			0.131
No	122	10.7	
Yes	77	18.2	
	**Desire for More Children**		
		
	**Yes (n = 27)**	**No (n = 172)**	
		
Age in years^c^, Mean ± SD	29.0 ± 5.8	35.0 ± 5.6	<0.001^d^
Number of pregnancies, Mean ± SD	3.0 ± 1.8	5.2 ± 2.7	< 0.001^d^
Number of living children, Mean ± SD	1.9 ± 1.3	4.3 ± 2.5	< 0.001^d^
Number of non-bio children, Mean ± SD	1.0 ± 1.1	1.3 ± 1.2	0.163^d^

^a ^The p-values are based on chi-square test unless otherwise specified.

^b ^Dwelling quality variable was divided into three categories as low, medium and high based on the house floor, wall and roof structure (low: mud floor, mud/thatched walls and grass/thatched roof; medium: mud floor, mud/thatched walls and metal roof; high: cement/concrete/wood floor, walls of permanent materials and metal roof or any two out of three (i.e., floor, walls and roof) of this structure).

^c ^The percentage who wants more children for age group 18-24, 25-29, 30-34, 35-39 and 40-44 years is 42, 30, 18, 4 and 4, respectively.

^d ^Two independent sample t-test p-value.

Use of effective family planning methods such as oral contraceptives and injectables was generally low in our respondents, however there was a high rate of condom use. Participants on HAART reported a much lower rate of condom use. For more details on contraceptive use see Table 2:

**Table 2:** Contraceptive use stratified for sex and HAART treatment status of survey respondents (n = 199)

	n (%)		n (%)	
	
	Female (n = 122)	Male (n = 77)	No HAART (n = 122)	HAART (n = 77)
OC Pill	4 ( 3.3)	2 ( 2.6)	3 ( 2.5)	3 ( 3.9)
Injectable	5 ( 4.1)	5 ( 6.5)	4 ( 3.3)	6 ( 7.8)
Male - Condom	67 (54.9)	47 (61.0)	87 (71.3)	27 (35.1)
Dual Method	-	7 ( 9.1)	5 ( 4.1)	2 ( 2.6)
Other	10 ( 8.2)	1 ( 1.3)	5 ( 4.1)	6 ( 7.8)
Missing/No Method	36 (29.5)	15 (19.5)	18 (14.8)	33 (42.9)

### Fertility desires

Overall, it was found that 13.6% of all participants wanted to have more children. HIV- positive individuals on HAART were more likely to want more children; however, this difference was not statistically significant (18.2% on HAART vs. 10.7% not on HAART, p = 0.131). Those on HAART who had two or more living children showed a trend to want to continue childbearing than those who were not on HAART (18.2% vs. 7.9%, p = 0.380). Those who wanted to continue childbearing wanted an average of 2.3 additional children (2.1 for participants on HAART vs. 2.4 for participants not on HAART). Three out of 14 pregnant women (21.4%) and 13 out of 132 non-pregnant women (9.9%) wanted more children (p = 0.187). For all participants, the average number of children desired (completed family size) was 4.3 with 3.7 children for participants on HAART vs. 4.6 children for participants not on HAART (p = 0.009).

In bivariate analysis, respondents who were on HAART were slightly more likely to say that they wanted more children in future (OR 1.86, p = 0.135), and this trend disappeared in the multivariate model. Results from the multivariate logistic regression analysis indicate that the odds of desiring more children were quite similar and not statistically different for those on HAART compared to those not on HAART. The final logistic regression model was adjusted for age, sex, occupation, and number of living children. Bivariate and multivariate logistic regression analysis results are shown in Table 3:

**Table 3:** Odds ratio (OR) and 95% confidence interval (95%CI) for a dependent variable "desire to have more children" (n = 199) - Logistic Regression Bivariate and Multivariate Analysis

	Bivariate Analysis		Multivariate Analysis	
	
Variable	OR (95% CI)	P	OR (95% CI)	P
Being on HAART				
No	1.00 (reference)		1.00 (reference)	
Yes	1.86 (0.82-4.21)	0.135	1.08 (0.40-2.90)	0.886
Age in years	0.84 (0.77-0.91)	< 0.001	0.89 (0.81-0.97)	0.012
Sex				
Female	1.00 (reference)		1.00 (reference)	
Male	1.86 (0.82-4.21)	0.135	3.01 (1.10-8.22)	0.032
Marital status				
Married	1.00 (reference)			
Cohabiting	1.07 (0.47-2.45)	0.870		
Occupation				
Farmer/peasant	1.00 (reference)		1.00 (reference)	
Businessperson	1.85 (0.61-5.66)	0.276	3.28 (0.82-13.1)	0.092
Others	1.61 (0.61-4.26)	0.341	2.39 (0.76-7.51)	0.137
Education				
None	1.00 (reference)			
Lower primary	0.65 (0.20-2.11)	0.471		
Upper primary	0.97 (0.12-2.83)	0.952		
Secondary and above	0.38 (0.07-2.07)	0.266		
Ethnic group				
Mutooro	1.00 (reference)			
Mukiga	1.95 (0.74-5.12)	< 0.001		
Other	3.55 (1.26-9.98)	0.016		
Religious affiliation				
Catholic	1.00 (reference)			
Protestant	1.38 (0.57-3.33)	0.473		
Muslim	1.05 (0.12-9.37)	0.964		
Other	1.10 (0.28-4.33)	0.887		
Dwelling quality				
Low	1.00 (reference)			
Medium	0.50 (0.15-1.67)	0.259		
High	0.30 (0.05-1.84)	0.192		
Pregnant				
No	1.00 (reference)			
Yes	2.50 (0.62-10.1)	0.200		
Number of living children	0.50 (0.38-0.68)	< 0.001	0.56 (0.39-0.80)	0.002
Number of non-bio children				
0	1.00 (reference)			
1	1.05 (0.34-3.23)	0.938		
2	0.87 (0.31-2.49)	0.798		
3	0.36 (0.10-1.36)	0.132		

A younger age, male sex, occupation as businessperson, and a smaller number of living children were significant predictors for the desire to have more children.

A sub-analysis was conducted to compare the fertility desires of men and women separately as fertility intentions of each sex may differ due to the diverse roles that men and women play in fertility decision-making and childbearing (Table 4).

**Table 4:** Odds ratio (OR) and 95% confidence interval (95%CI) for a dependent variable "desire to have more children" (n = 199) by sex - Logistic Regression Multivariate Analysis

	Males (n = 77)		Females (n = 122)	
	
Variable	OR (95% CI)	P	OR (95% CI)	P
Being on HAART				
No	1.00 (reference)		1.00 (reference)	
Yes	1.88 (0.46-7.73)	0.380	0.65 (0.13-3.32)	0.605
Age in years	0.85 (0.73-0.99)	0.031	0.91 (0.80-1.05)	0.187
Occupation				
Farmer/peasant	1.00 (reference)		1.00 (reference)	
Businessperson	2.49 (0.43-14.5)	0.311	11.2 (0.86-147.1)	0.066
Others	0.55 (0.05-5.51)	0.608	10.3 (1.46-72.7)	0.019
Number of living children	0.70 (0.43-1.14)	0.149	0.35 (0.18-0.70)	0.003

The main result of the sub-analysis was that being on HAART was not a predictor for fertility desires of males or females. However, the direction of the adjusted OR was different; men on HAART had a tendency to desire more children whereas women on HAART tended to desire fewer children than their untreated counterparts. Additionally, a higher number of living children was a statistically significant predictor for a lower desire for more children in women (OR 0.35, 95% CI 0.18-0.70). This association was the same in men, but did not reach statistical significance. We also found a significant association between the desire to have more children and the occupational status as well as the number of living children for females, but not for males (see Table 3).

### Knowledge and risk perceptions of mother-to-child-transmission of HIV

Participants were asked questions to assess their knowledge of MTCT of HIV, including whether or not being on HAART would influence perceptions of mother-to child transmission. Many respondents said that it depended upon whether the woman delivered her baby in the village or at the health centre/hospital. Participants were asked if MTCT of HIV was possible given differing treatment and delivery scenarios (see Table 5).

**Table 5:** Knowledge and risk perception of mothers on HAART and not on HAART

	% who responded that MTCT of HIV is likely under the circumstances	
	
	On HAART*	Not on HAART*
If mother not on anti-retroviral treatment	92.6	96.2
If mother on anti-retroviral treatment	42.3	44.4
If mother not on anti-retroviral treatment and gives birth in village	100.0	100.0
If mother on anti-retroviral treatment and gives birth in village	76.5	67.6
If mother not on anti-retroviral treatment and gives birth in hospital	52.3	45.2
If mother on anti-retroviral treatment and gives birth in hospital	15.3	10.5

* Responses of the participants were not significantly different (i.e., p > 0.05) for those on HAART vs. not on HAART

The results from Table 5 show that participants generally knew being on HAART reduces the risk of MTCT. A majority of respondents said that women on HAART cannot transmit HIV to their unborn/newborn child (the correct answer would have been that the MTCT risk is minimal). Most respondents stated that the birth setting in the hospital (as compared to a home birth usually attended by traditional birth attendants), was a preferred place in order to reduce MTCT as hospital/health centre deliveries were perceived to be more sanitary and were assisted by trained medical personnel. Participants tended to think that the birth setting was more important than the mother's HAART status. HIV-positive participants on HAART and those not on HAART gave more or less the same responses. Answers to all questions did not differ statistically based upon the respondents' HAART status, indicating that the MTCT knowledge and risk perception of HIV in both groups was the same.

The questionnaire also assessed the attitude towards childbearing in HIV-positive women/couples. Generally, most participants were not in favor of having children when one or both of the parents are HIV-infected (90.9% of participants on HAART vs. 94.4% of participants not on HAART, p = 0.496). They also thought that their community members had a negative attitude towards HIV-infected couples when they had a newborn baby.

## Discussion

This cross-sectional study conducted in western Uganda included HIV-positive respondents on HAART and not on HAART in order to elucidate their fertility desires as a result of their HIV treatment status. Importantly, this study included men and women which enabled us to assess gender differences when the variable "fertility desires" in conjunction with HAART was modeled for men and women separately. Several other studies have reviewed only fertility desires in (pregnant) women thus missing the male partners' perspectives on fertility desires. These studies limited their interviews to women perhaps because access to interviewing women is easier and also because traditionally childbearing is generally associated with the female gender [18,19,29,30].

Our study contributes to expanded knowledge on the relationship between HAART treatment status and the desire for children. We did not find that HAART treatment status had an impact for the desire for children. It is important for the scientific community and health care workers to know this. It is also important to assess the level of knowledge on the relationship between MTCT and HAART in health care workers who are working in HIV and family planning programs. They should be aware of the impact of HAART on fertility desires (and on possible negative maternal pregnancy outcomes) in order to provide updated, balanced and effective counselling for family planning and HIV care and prevention program clients.

Our main study finding was that we did not detect an association between being on HAART and an increased desire for future children. Therefore, it is not likely a consideration by HIV-infected persons and couples in their discussions about planning and achieving their desired family size. This corresponds to what Homsy et al. found in their study from eastern Uganda [19] and what Snow described from Mbarara [20].

Males were generally more likely than females to want more children which we expected and which is in accordance with the literature from sub-Saharan Africa. Other predictors for a higher fertility desire (see Table 3) were younger age, higher number of living children, and higher occupational status (only for women), which are consistent with other findings from the literature for sub-Saharan Africa. In the gender-specific sub-analysis, men on HAART had a tendency to desire more children compared to those men not on HAART, while women on HAART had less desire for future children compared to women not on HAART (both associations were not statistically significant). However, these results from the sub-analysis have to be considered with caution, as the 95% confidence intervals were very wide and difficult to interpret. In addition, the sample size in this sub-analysis was small and may not provide the statistical power to detect a statistically significant difference. The gender differences with respect to the direction of the relationship between HAART treatment and fertility desires should be tested in larger studies with sufficient power. If confirmed, it would be important for professionals to use different approaches in counseling male and female persons on their fertility options when they are HIV-positive. It would be also important to confirm if women on HAART have less desire for future children than women not on HAART and, if so, why.

The non-association between fertility desires and being on HAART treatment in HIV-infected rural Ugandans is not too surprising, as poor rural Ugandans with low educational attainment do not base their life decisions on the probabilities of HIV transmission but rather on life experiences. The hypothesis that their fertility desires are based on the probability of mother-to-child transmission of HIV seems therefore counterintuitive, as many other factors may play a role in fertility desires. These factors could be whether the parents will be alive to see the child grow and if they will be well enough to care for the child. If parents are receiving HAART, we can assume that they were quite sick (stage 3 or 4 or CD+ count of less than 200 cells/mm^3 ^[31]. This would suggest that at some point in time they have experienced some of the more challenging aspects of living with HIV such as morbidity and adverse reactions to HAART treatment regimens. These experiences might have reduced their fertility desires. In addition, the provision of HAART in Uganda is not guaranteed, making the choice to have children especially risky for the group relying on HAART for their well being [32]. Also, the risk of vertical HIV transmission for mothers on HAART is not zero and so a small risk remains.

It was surprising to find that the knowledge about vertical transmission of HIV and its risk reduction in mothers on HAART did not differ whether participants were on HAART or not. We expected participants in the HAART group to be more knowledgeable, as there is the duty for the HAART clinic staff to provide the proper and updated information to their clients. This lack of knowledge is regrettable, as the HAART clinic staff should have had many opportunities as a result of their treatment program (especially during the monthly monitoring visits) to properly inform their clients.

Reproductive decision-making may change if women/couples receive HAART for a longer time period. We collected our data in 2006 amidst a recent and rapid expansion of available free HAART services in Uganda. As we did not ask the question of the duration of HAART treatment in our survey, we were unable to incorporate this variable in our statistical models. It has been suggested that the duration of HAART treatment could play a role in the association between HAART status and fertility desires, e.g. women on HAART treatment for a longer time may be more inclined to have more children [21]. Future studies may want to include patients with extended HAART experience to elucidate their fertility desires.

### Limitations

Our study had some limitations. First, the odds ratios were based on cross-sectional data which precludes assessing the causality of the associations described. Second, social desirability bias cannot be excluded as the information collected was sensitive. However, we used highly trained and experienced interviewers to minimize this bias. Third, we may have experienced a selection bias as we were not able to perform a true random sampling procedure. The fourth limitation was that we were not able to assess any clinical parameters in regard to the HIV disease progression of participants. As some participants on HAART may have been in a more advanced clinical stage of HIV, they may be less likely wanting more children in future due to their reduced physical health status. If this is true, we may have overstated the non-association between HAART and fertility desires for more children. Finally, as free HAART was made available in Uganda in 2005, it was likely that some of our participants were put on HAART during this expansion and therefore had a short duration of antiretroviral treatment when they were interviewed by us. Another shortcoming is that this study was conducted in 2006; since then there have been changes in HAART promotion and its use in Uganda, the results should be interpreted with caution.

In addition to interviewing participants about the desire for children, we also assessed the pregnancy status at the time of the survey by asking the respondents if they (or their partners) were pregnant. The proportion for those on HAART who reported to be pregnant at the time of the interview were similar compared to those respondents (or their partners) not on HAART at the time of the interviews. However, as the absolute numbers were very small, the results from this comparison have limitations. In spite of these limitations, these pregnancy numbers somewhat validate the findings from the interviews.

## Conclusions

In order to provide updated, complete, comprehensive and balanced information during counseling and treatment sessions to HIV-infected persons/couples ethically, counseling staff has the responsibility to inform clients not only of the benefits that can be conferred by HAART but also of the possible risks for negative pregnancy outcomes [33,34]. There should be no delay in relaying the complete educational package to this group of HIV-positive persons and couples, as well as to the population in general. HIV counseling, care and prevention services as well as family planning programs are still not adequately addressing the benefits of HAART. The package should be modified to include current and updated information re: the effectiveness of HAART on reducing vertical transmission of HIV. Health care workers and counselors who provide HIV/family planning counseling should upgrade their MTCT/HAART knowledge and be required to provide this information to all service users in an unbiased way. Service providers should be evaluated on their practice of providing this vital, relevant and updated information to their clients. Evidence on the positive impact of HAART on vertical HIV transmission is crucial for the HIV-positive persons/couples to know when they are making childbearing decisions.

## Competing interests

The authors declare that they have no competing interests.

## Authors' contributions

WK was involved in all stages of the study and wrote the article. JH had major input in the development of the proposal and conducted the field work in Uganda. She commented on the draft manuscript and helped with the interpretation of the study results. GSJ developed the statistical part of the proposal and analyzed the data. He also provided input into the manuscript. AA helped with the supervision of the field work and provided input into the manuscript and the interpretation of the data. TR was involved in the design of the study and supervised the field work in Uganda. He also provided input into the manuscript and helped to interpret the study results within the context of the Ugandan health care system. All authors read and approved the final manuscript.
